# Rayleigh-scattering microscopy for tracking and sizing nanoparticles in focused aerosol beams

**DOI:** 10.1107/S2052252518010837

**Published:** 2018-09-11

**Authors:** Max F. Hantke, Johan Bielecki, Olena Kulyk, Daniel Westphal, Daniel S. D. Larsson, Martin Svenda, Hemanth K. N. Reddy, Richard A. Kirian, Jakob Andreasson, Janos Hajdu, Filipe R. N. C. Maia

**Affiliations:** a Chemistry Research Laboratory, Department of Chemistry, Oxford University, 12 Mansfield Rd, Oxford OX1 3TA, UK; b Laboratory of Molecular Biophysics, Department of Cell and Molecular Biology, Uppsala University, Husargatan 3 (Box 596), Uppsala SE-75124, Sweden; c European XFEL GmbH, Holzkoppel 4, Schenefeld 22869, Germany; d Institute of Physics, ELI Beamlines, Academy of Sciences of the Czech Republic, Na Slovance 2, Prague CZ-18221, Czech Republic; e Department of Physics, Arizona State University, 550 E. Tyler Drive, Tempe, AZ 85287, USA; f Condensed Matter Physics, Department of Physics, Chalmers University of Technology, Gothenburg, Sweden; g NERSC, Lawrence Berkeley National Laboratory, Berkeley, California, USA

**Keywords:** Rayleigh scattering, XFELs, aerosol injection, Uppsala injectors, nanoparticles

## Abstract

Ultra-bright femtosecond X-ray pulses generated by X-ray free-electron lasers (XFELs) can be used to image high-resolution structures without the need for crystallization. For this approach, aerosol injection has been a successful method to deliver 70–2000 nm particles into the XFEL beam efficiently and at low noise. Improving the technique of aerosol sample delivery and extending it to single proteins necessitates quantitative aerosol diagnostics. Here a lab-based technique is introduced for Rayleigh-scattering microscopy allowing us to track and size aerosolized particles down to 40 nm in diameter as they exit the injector. This technique was used to characterize the ‘Uppsala injector’, which is a pioneering and frequently used aerosol sample injector for XFEL single-particle imaging. The particle-beam focus, particle velocities, particle density and injection yield were measured at different operating conditions. It is also shown how high particle densities and good injection yields can be reached for large particles (100–500 nm). It is found that with decreasing particle size, particle densities and injection yields deteriorate, indicating the need for different injection strategies to extend XFEL imaging to smaller targets, such as single proteins. This work demonstrates the power of Rayleigh-scattering microscopy for studying focused aerosol beams quantitatively. It lays the foundation for lab-based injector development and online injection diagnostics for XFEL research. In the future, the technique may also find application in other fields that employ focused aerosol beams, such as mass spectrometry, particle deposition, fuel injection and three-dimensional printing techniques.

## Abstract 2

Rayleigh-scattering microscopy permits tracking and sizing of aerosolized particles down to 40 nm in diameter. It lays the foundation for lab-based injector development and online injection diagnostics for X-ray free-electron laser (XFEL) research.

## 1. Introduction   

Extremely intense and short X-ray free-electron laser (XFEL) pulses can outrun processes of radiation damage (Neutze *et al.*, 2000 ▸) and the short pulse duration permits, in principle, solving structures at room temperature without the requirement for crystallization (Seibert *et al.*, 2011 ▸). XFEL single-particle imaging has been demonstrated on relatively large samples (70–2000 nm) to moderate resolutions (Seibert *et al.*, 2011 ▸; Hantke *et al.*, 2014 ▸; Ekeberg *et al.*, 2015 ▸). With continued improvements to sample delivery techniques, XFEL beam intensity, beamlines, detectors and reconstruction algorithms, the XFEL single-particle imaging technique has the potential to generate structures at high acquisition rates (Hantke *et al.*, 2014 ▸) with particle sizes ranging from several microns (for example, entire cells) (Bergh *et al.*, 2008 ▸) to a few nanometres (for example, single proteins) (Neutze *et al.*, 2000 ▸). In addition, new strategies have been proposed that make use of inelastic photoemission and would allow chemically selective imaging at atomic resolution (Classen *et al.*, 2017 ▸).

Despite the extremely bright illumination attainable with today’s most powerful XFELs, a small particle, such as a single protein, only gives rise to a faint and noisy diffraction pattern (Neutze *et al.*, 2000 ▸). Signal averaging over many identical particles is needed to reconstruct the high-resolution structure.

Efficient sample delivery with low background noise is central to the success of the approach. Substrate-based sample delivery for XFEL single-particle imaging of biological samples has been demonstrated (Seibert *et al.*, 2010 ▸; Kimura *et al.*, 2014 ▸) but the presence of a sample container or substrate is a source of background noise that must be avoided when aiming for atomic resolution. Atomically thin substrates such as graphene could potentially solve this problem. Nevertheless, contact to any substrate typically affects structure and orientation of the deposited sample (Zeng *et al.*, 2017 ▸). Moreover, sample exchanges within less than a microsecond are required to take full advantage of the rapid repetition rates of modern XFELs, and this seems unfeasible with substrate-based techniques and could be challenging with liquid jets (Stan *et al.*, 2016 ▸). Aerosol sample delivery lifts the requirement for any sample support, which significantly reduces background scattering and allows for data collection at high rates (Bogan *et al.*, 2008 ▸; Seibert *et al.*, 2011 ▸; Hantke *et al.*, 2014 ▸). While aerosol sample delivery is in principle an elegant approach with attractive advantages, it requires an aerosol injector that reaches high particle densities for achieving high hit ratios (*i.e.* fractions of XFEL pulses that hit at least one particle) and sufficient particle speed to prevent multiple exposures.

**Figure 1:**
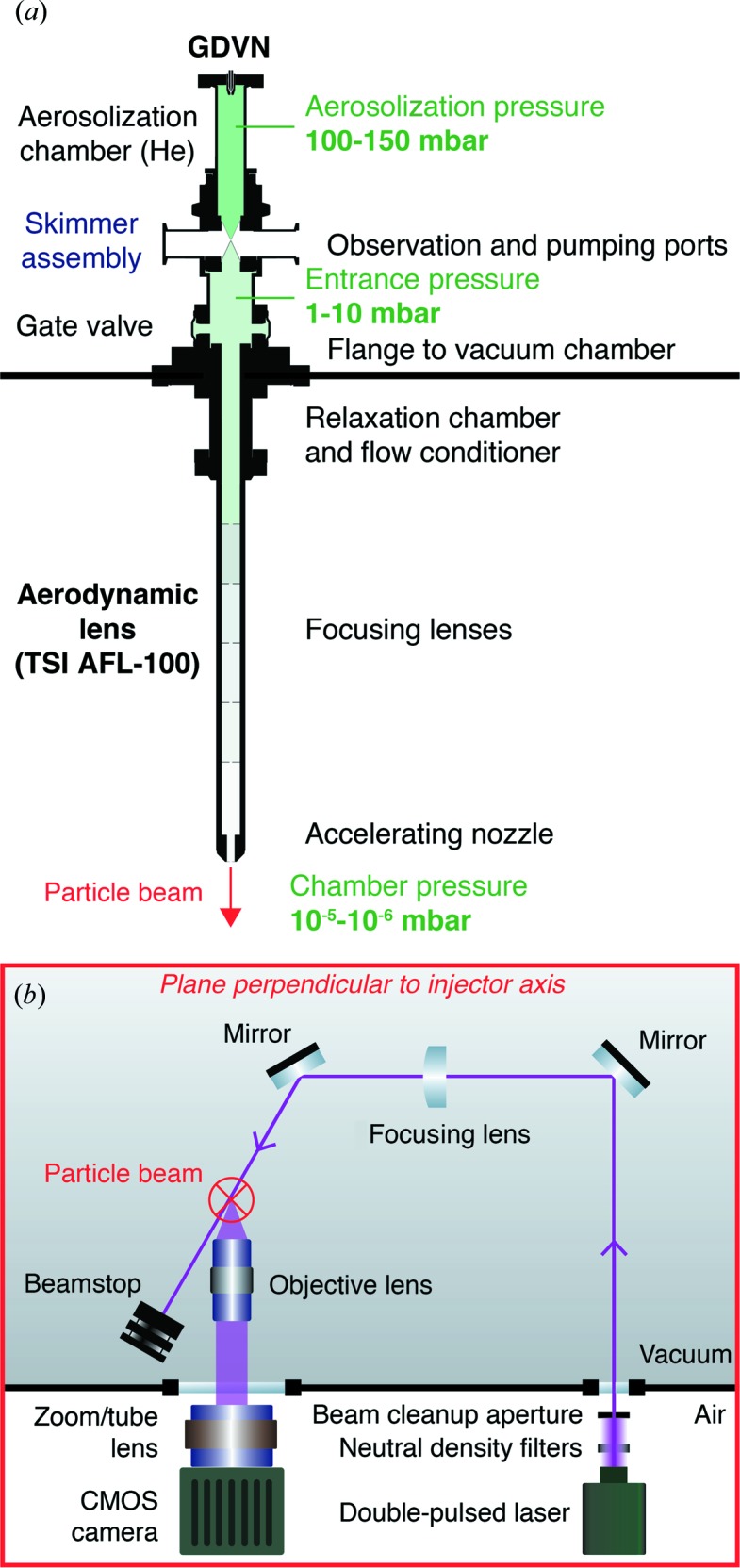
Experimental setup. (*a*) Section of the Uppsala injector along the particle-beam axis. The injector is equipped with a gas dynamic virtual nozzle (GDVN) for sample aerosolization, a skimmer for excess-gas removal and an aerodynamic lens for particle-beam focusing. (*b*) Schematic of the Rayleigh-scattering-microscopy setup. The optical beam path is confined to the plane perpendicular to the injector axis [view perpendicular to image plane in (*a*)].

A pioneering aerosol injector for XFEL single-particle imaging, the ‘Uppsala injector’ (Seibert *et al.*, 2011 ▸; Hantke *et al.*, 2014 ▸) (Fig. 1 ▸ *a*), has demonstrated success in numerous experiments (Seibert *et al.*, 2011 ▸; Rath *et al.*, 2014 ▸; Hantke *et al.*, 2014 ▸; van der Schot *et al.*, 2015 ▸; Ekeberg *et al.*, 2015 ▸; Reddy *et al.*, 2017 ▸) for particles between 70 and 2000 nm in diameter. The injector is available for users at the Linac Coherent Light Source (LCLS), the European XFEL, the Free Electron laser Radiation for Multidisciplinary Investigations (FERMI) and the Extreme Light Infrastructure (ELI) Beamlines facility. Despite its successful and frequent use, the particle-beam properties as a function of operating conditions that are relevant for XFEL single-particle imaging have not yet been extensively characterized and described in the literature.

Traditionally, focused-aerosol-particle beams have been examined on the basis of dusting spots (Murphy & Sears, 1964 ▸; Williams *et al.*, 2013 ▸) or, in the case of charged aerosols, using ion detectors (Schreiner *et al.*, 1999 ▸; Williams *et al.*, 2013 ▸). Recently, visualization of relatively large (down to 200 nm) aerosol-injected particles has been demonstrated (Kirian *et al.*, 2015 ▸; Awel *et al.*, 2016 ▸, 2018 ▸). Here we describe a Rayleigh-scattering-microscopy setup (Fig. 1 ▸ *b*) that extends the size range of this approach down to 40 nm in diameter and that can be used to directly measure positions, velocity and, as an additional quantity, the diameter of single aerosol particles.

We used our Rayleigh-microscopy setup to characterize the particle-beam properties of the Uppsala injector. We present results on particle focusing, velocity, particle density and overall injection yield as functions of operating conditions. We discuss implications of our results for XFEL single-particle imaging and strategies for future injector development.

## 2. Results   

### 2.1. Experimental setup   

The Uppsala injector (Fig. 1 ▸ *a*) is composed of an aerosolization chamber, a nozzle/skimmer stage for excess-gas removal (Campargue, 1984 ▸; Beijerinck *et al.*, 1985 ▸) and an aerodynamic lens (Murphy & Sears, 1964 ▸; Bogan *et al.*, 2008 ▸; Liu *et al.*, 1995*a* ▸,*b* ▸) for aerosol focusing at the XFEL beam focus [usually 0.1 to several tens of microns in diameter (Boutet & Williams, 2010 ▸; Bostedt *et al.*, 2013 ▸; Feldhaus, 2010 ▸)]. The pressure decreases gradually as the particle-laden gas flows through the injector compartments. In the first compartment the sample solution is aerosolized with a gas dynamic virtual nozzle (GDVN) (Gañán-Calvo, 1998 ▸; DePonte *et al.*, 2008 ▸) in a 100–250 mbar He atmosphere. After aerosolization, excess gas is skimmed away by differential pumping in the nozzle-skimmer stage. Downstream of the skimmer the aerosol particles enter the aerodynamic lens at a pressure between 0.5 and 3.5 mbar (aerodynamic lens entrance pressure). Particles exit the aerodynamic lens through an acceleration tube and a 1.5 mm aperture, and enter the experimental chamber, which is kept at 10^−6^–10^−4^ mbar (vacuum-chamber pressure).

**Figure 2:**
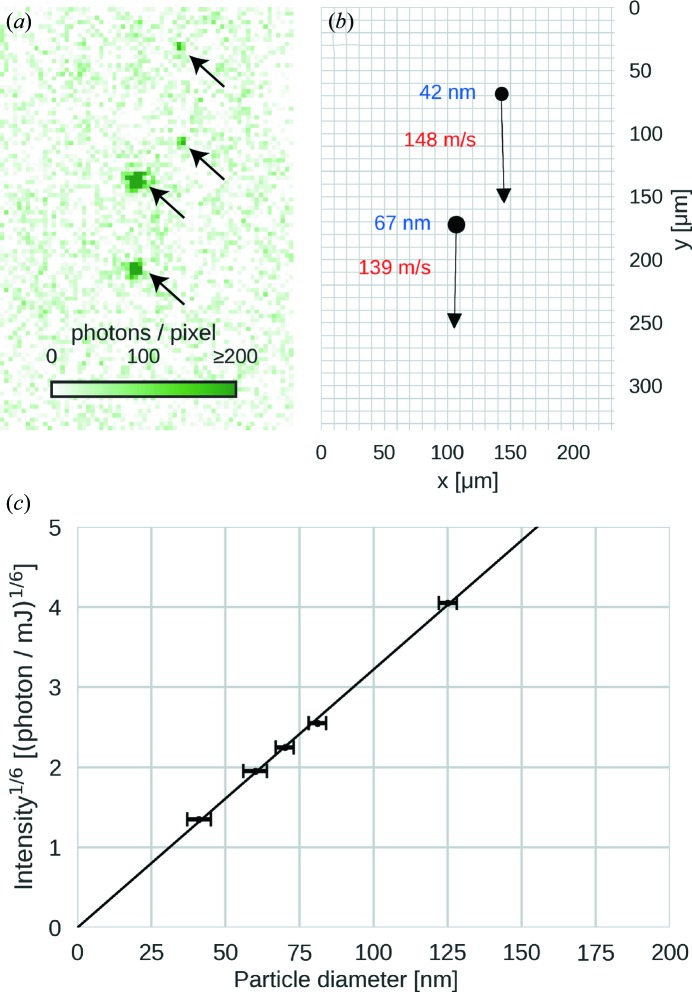
Quantitative analysis of Rayleigh-scattering-microscopy data. (*a*) Double-exposure image of two polystyrene spheres of different diameters. The pulse delay was 50.8 µs and the pulse energy 56.1 mJ. (*b*) Extracted particle positions, velocities and diameters from the image shown in (*a*). (*c*) The sixth root of the mean integrated scattering intensity per particle (rescaled to 1 mJ laser-pulse energy) is plotted against the diameter of the respective polystyrene-sphere size standard. The values follow Rayleigh’s scattering law (solid line).

A double-pulsed green laser illuminates particles as they exit the injector. Images are taken with a microscope equipped with a CMOS camera (Fig. 1 ▸ *b*). The double-flash illumination results in two particle images per exposure. Velocities are determined from the relative distances and the inter-pulse delay (Fig. 2 ▸ *a*). The data are analyzed with our open-source software package (https://github.com/mhantke/spts), which determines particle positions, velocities and particle diameters from the images (Figs. 2 ▸ *a* and 2*b*).

With two laser pulses delayed by 0.5 µs the lateral extent of the laser-beam spot [*i.e.* 0.5 mm full width at half-maximum (FWHM)] permits, in principle, the measurement of velocities up to 1000 ms^−1^. The injector points downwards into the experimental chamber while the path of the laser beam and the optical axis of the microscope are confined to the horizontal plane. The optical axis of the microscope intersects the laser-beam axis at an angle of 25°. Generally, the Mie-scattering law can be used to estimate the particle brightness as a function of particle diameter (Bohren & Huffman, 1983 ▸). In this small-angle scattering configuration the particles with diameters up to 200 nm can be considered as Rayleigh scatterers and the scattering intensity is proportional to the sixth power of the particle diameter. We confirmed this scaling law for our setup by measuring the particle brightness in images of polystyrene-sphere size standards (Fig. 2 ▸ *c*). The Rayleigh-scattering intensity increases monotonically with particle diameter. This means that, if suitable calibration data (as shown in Fig. 2 ▸ *c*) are available, diameters of particles of unknown size may be determined from the measured particle brightness in the image. For a 40 nm polystyrene sphere we measured an average scattering intensity of 157 photons per 50 mJ pulse with an optical system of 0.055 numerical aperture. Attempts at imaging even smaller 20 nm sized injected polystyrene spheres failed at this pulse energy. This was expected as their scattering signal is 64 times lower than for the 40 nm spheres (three photons per particle), not exceeding average background fluctuations (four photons per pixel). Particles larger than 125 nm could not be quantitatively sized because of the limited linear dynamic range of the detector. These are technical limitations, which could be overcome by a tighter laser focus, higher pulse energies, a larger numerical aperture for the objective lens and a higher dynamic range for the detector.

### 2.2. Particle-beam focusing   

**Figure 3:**
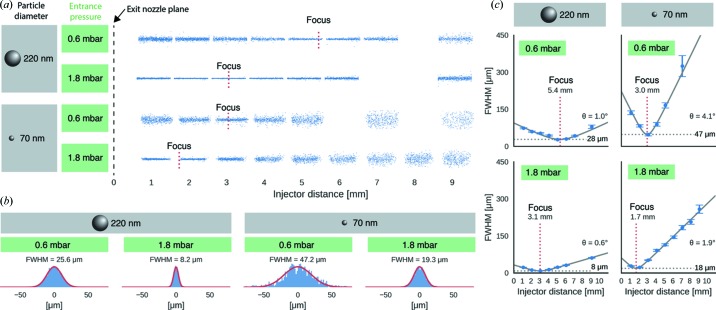
Particle-beam focusing as function of entrance pressure and particle diameter. (*a*) Blue dots represent measured particle positions of injected polystyrene spheres (70 nm and 220 nm in diameter) at entrance pressures of 0.6 mbar and 1.8 mbar, respectively. Gaps are a result of combining the data from measurements at fixed injector distances without overlap of the fields of view. The positions of the focus planes are indicated by dotted red lines. (*b*) Measured particle-beam profiles (blue histograms) in the particle-focus plane were approximated by Gaussian functions (red lines). (*c*) The evolution of the particle-beam width (blue circles) was approximated with a Gaussian-beam model (black solid lines). The model is parameterized by a divergence angle θ, the beam waist (gray dashed lines) and the position of the focus plane (red dotted lines).

**Figure 4:**
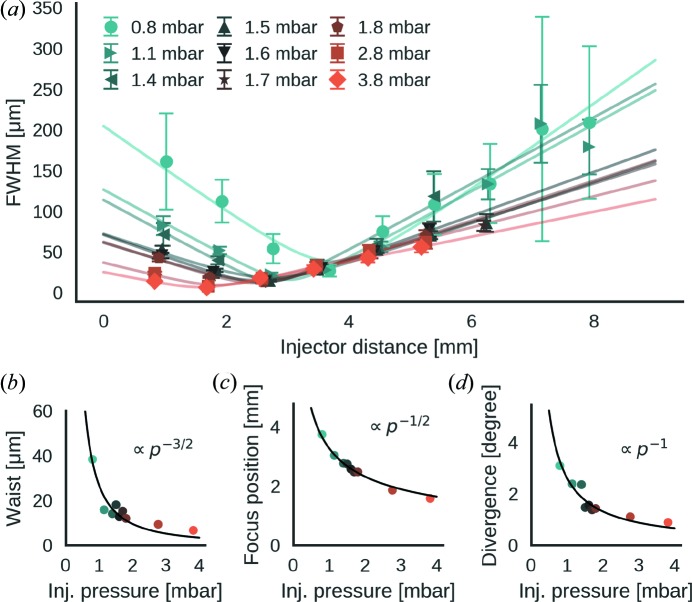
Scaling laws for the particle-beam focus with respect to entrance pressure. (*a*) Particle-beam widths (FWHM) for a range of pressures (see legend) are plotted against the distance from the injector tip. Particles were polystyrene spheres of 100 nm in diameter. The experimental data can be approximated with a Gaussian-beam model (solid lines). Scaling laws [solid lines in (*b*), (*c*) and (*d*)] were identified for the model parameters as functions of the entrance pressure *p*. (*b*) The particle-beam waist scales as *p* ^−(3/2)^. (*c*) The distance between particle focus and injector tip scales as *p* ^−(1/2)^. (*d*) The particle-beam divergence scales as *p* ^−1^.

To study the particle-beam evolution as a function of particle diameter and entrance pressure of the aerodynamic lens we recorded separate data sets for polystyrene spheres with mean diameters between 40 and 495 nm. Data were collected for about 1.5 min at a frame rate of 15 Hz resulting in about 1000 images per data set. We observed one to 30 particles per frame depending on particle size, particle concentration, injector pressure and distance from the exit orifice of the aerodynamic lens. The field of view was confined to the illumination spot. To examine the particle-beam evolution, we translated the injector along the particle-beam axis and measured recorded data for a range of entrance pressures, particle diameters and distances from the injector tip (‘injector distance’) (Fig. 3 ▸ *a*). Particle clusters, caused by statistical variation of the occupancy of droplets generated by the nebulizer, were identified by their larger particle diameter and excluded from the analysis (see Fig. S1 in the supporting information). The data show that for a given particle size an increase in entrance pressure results in a contraction of the beam profile (Figs. 3 ▸ *a* and 3*b*). As the particle size or entrance pressure increases, the particle beam became tighter, less divergent, and its focus (here defined as the location of minimum beam diameter) moved closer to the injector tip (Fig. 3 ▸ *c*). At any given injector distance, the particle-density profile transverse to the beam direction followed a Gaussian distribution (Figs. 3 ▸ *b* and S1 in the supporting information) and the evolution of the particle beam as a function of injector distance was well approximated by a Gaussian-beam model (Figs. 3 ▸ *c* and 4 ▸ *a*). For a given particle diameter an increase in injector pressure *p* resulted in a tighter beam waist as *p* ^−(2/3)^ (Fig. 4 ▸ *b*), a reduction of the distance between beam focus and injector tip as *p* ^−(1/2)^ (Fig. 4 ▸ *c*), and a decrease in particle beam divergence as *p* ^−1^ (Fig. 4 ▸ *d*).

### 2.3. Particle speed and acceleration   

**Figure 5:**
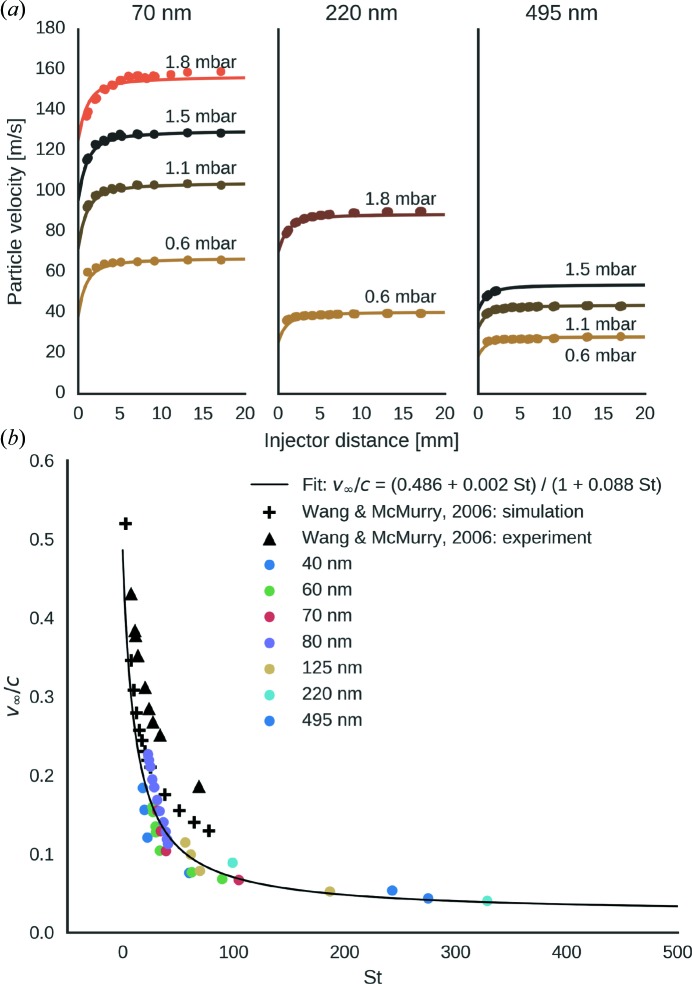
Particle speed and acceleration. (*a*) Particle velocity as a function of their distance from the exit orifice of the injector for polystyrene spheres of 70, 220 and 495 nm in diameter at pressures between 0.6 and 1.8 mbar. The solid lines show the approximated velocity evolution according to our model. (*b*) Terminal-velocity values normalized to the speed of sound plotted against the Stokes number. We compare our data (filled circles) to simulated and experimental data reported by Wang & McMurry (2006 ▸) for the same lens system with air as a carrier gas and at higher injector pressures than studied here.

At the exit of the injector nozzle the gas expands as a free jet into the experimental chamber and forms a divergent flow field that accelerates the aerosol particles. Fig. 5 ▸(*a*) shows the measured values for velocities of injected polystyrene spheres with mean diameters of 70, 220 and 495 nm at a range of injector pressures. The data can be approximated (solid lines in Fig. 5 ▸ *a*) by combining two semi-empirical models that describe flow dynamics of the gas (Ashkenas & Sherman, 1966 ▸; Dahneke & Cheng, 1979 ▸) and the drift forces for spherical aerosol particles (Henderson, 1976 ▸). The entrance velocities and the ‘effective nozzle diameter’ were treated as free parameters and fitted to best match the data.

The dimensionless parameter that governs particle focusing by an aerodynamic lens is the Stokes number. We defined the Stokes number in accordance with Wang & McMurry (2006) ▸:
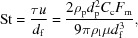
where *τ* denotes the particle relaxation time, *u* the average flow velocity at the lens orifice, *ρ*_p_ the particle-mass density, *d*_p_ the particle diameter, *C*_c_ the discharge coefficient, *F*_m_ the mass flow rate, *ρ*_l_ the mass density of the fluid, *μ* the dynamic viscosity and *d*_f_ the diameter of the exit orifice of the aerodynamic lens. For calculating the Stokes number for all flow conditions, we deduced values for *C*_c_, *F*_m_ and *ρ*_l_ from our numerical flow model. In Fig. 5 ▸(*b*) we plotted the measured terminal velocities normalized by the speed of sound (1008 ms^−1^ for He at standard conditions) against the Stokes number and observed that all measured velocities collapsed onto a single curve. The relation between Stokes number and velocity was fitted to the function (solid line) suggested by Wang & McMurry (2006) ▸:
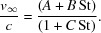
We obtain the best fit to our data for *A* = 0.486, *B* = 0.002 and *C* = 0.088, which roughly match data that Wang & McMurry (2006 ▸) reported for Stokes numbers below 100 (Fig. 5 ▸ *b*). Our data extends to higher Stokes numbers for which, to our knowledge, no data has been published that we could use for comparative purposes.

### 2.4. Particle densities   

**Figure 6:**
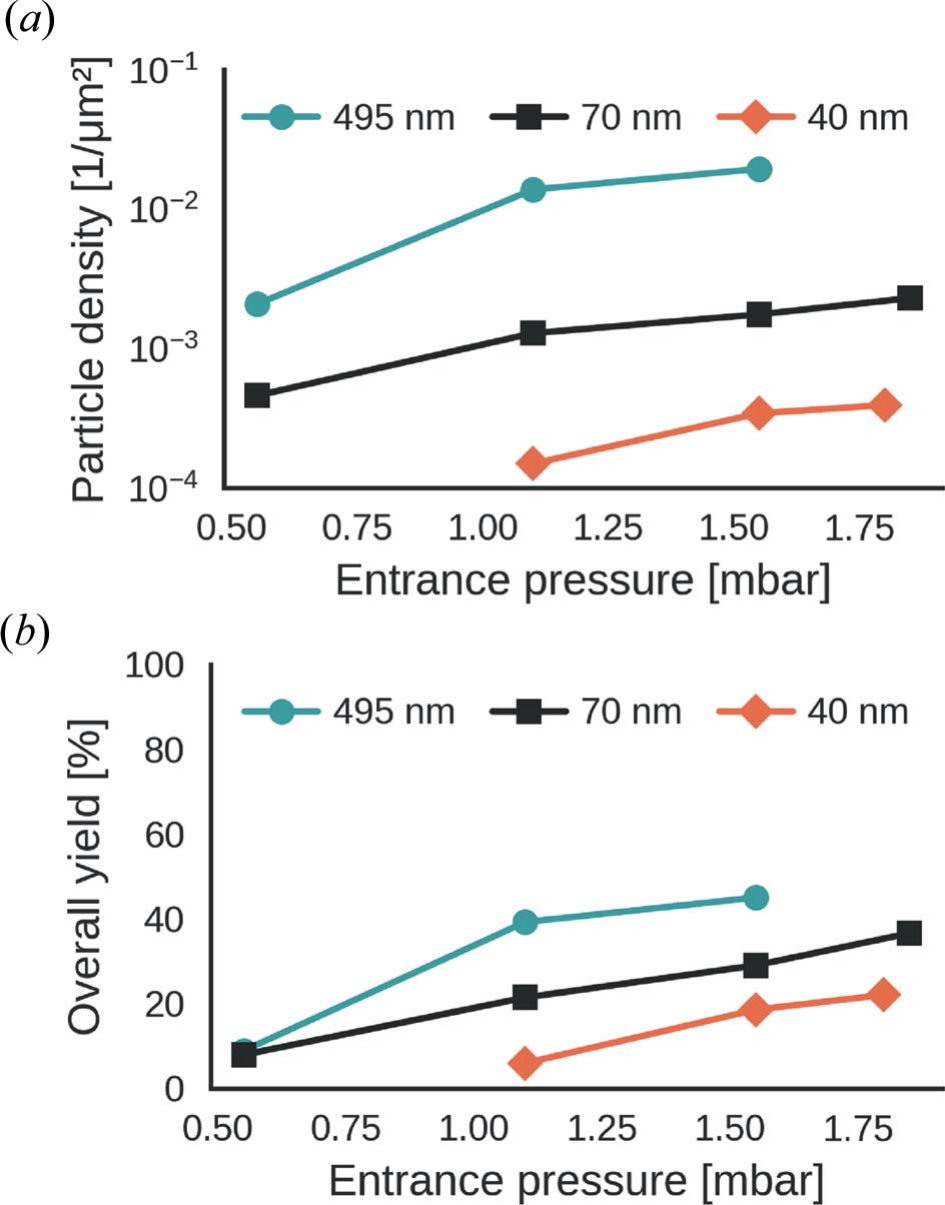
Particle density (*a*) and overall particle-injection yield (*b*) as a function of aerodynamic lens entrance pressure for polystyrene spheres with a range of distinct diameters (see legends). For (*a*) we normalized the values to the conditions of a particle solution with a concentration of 10^12^ particles per ml and a flow rate of 1 µl min^−1^.

We determined the particle density as a function of particle diameter and entrance pressure (Fig. 6 ▸ *a*) at a flow rate of 1 µl min^−1^ and a concentration of 10^12^ particles per ml. We found that for a given particle size between 40 and 495 nm the maximum areal particle-number density was in the range of 4 × 10^−4^ to 1.9 × 10^−2^ particles per µm^2^ (Fig. 6 ▸ *a*). By comparing the inflow of particles (particle concentration × sample flow rate) to the outflow from the injector (particle-number density × cross-sectional beam area × particle velocity) we determined the maximum particle-injection yield, from solution into vacuum, as 22 and 45% depending on particle diameter (Fig. 6 ▸ *b*).

Particles accelerate after they pass the last orifice and therefore the location of maximum particle density does not necessarily coincide with the focus position (*i.e.* the location of minimum beam diameter). We determined the areal particle-number density as a function of injector distance (see Fig. S2 in the supporting information) and found that the location of its maximum matches the position of the focus to the precision of our measurements.

## 3. Discussion   

In this work we introduced a lab-based technique that allows tracking and sizing of unlabeled aerosol particles as they are injected into a vacuum chamber. We applied this technique to characterize the Uppsala injector, which has been the pioneering sample injector for XFEL imaging. Our data enabled us to determine particle-beam characteristics as a function of particle diameter and aerodynamic lens entrance pressure.

### 3.1. Characterization of the aerodynamic lens   

We found that the particle-beam profile of the Uppsala injector is represented accurately by a Gaussian-beam model for the range of tested conditions (40–500 nm particle diameter, 0.5–2.0 mbar entrance pressure). An increase in entrance pressure and particle diameter results in a contraction of the beam profile. For a fixed particle diameter, the contraction is characterized by a tighter beam waist, shorter focus distance and lower particle-beam divergence, each governed by a simple scaling law of the entrance pressure.

We developed a numerical model to describe the acceleration of particles as they exit the aerodynamic lens. The model allowed us to assign a Stokes number to each measurement. Data points of final particle velocity plotted against the Stokes number collapse onto a single curve. The curve is in agreement with previous results (Wang & McMurry, 2006 ▸) and extends beyond. Our velocity measurements show that even the largest particles (500 nm) are fast enough (>20 m s^−1^) to pass through a 1 µm focus within the time gap between two X-ray pulses given the 4.4 MHz repetition rate of the European XFEL. This means that the particles are fast enough to clear the interaction volume between subsequent pulses. This permits, in principle, data collection at the theoretical maximum rate (Hantke *et al.*, 2014 ▸).

### 3.2. Particle size   

We showed that the brightness of single particles in our images follows the Rayleigh-scattering law. We demonstrated that, with a calibration curve, injected particles between 40 and 125 nm in diameter can not only be detected but also sized. This is particularly useful for XFEL single-particle imaging because the undesired presence of non-volatile contaminants or insufficient particle desolvation can be discovered by a mismatch in the size distributions of sample particles before and after aerosolization (Kassemeyer *et al.*, 2012 ▸; Daurer *et al.*, 2017 ▸). The ability to test in the lab injection of any given sample helps to identify suitable sample buffers and sample concentrations prior to data collection at XFELs. This information is essential for optimizing sample injection for XFEL single-particle imaging and difficult to obtain by other means.

### 3.3. Particle densities   

We showed that the particle densities that can be reached with the Uppsala injector depend significantly on particle diameter and entrance pressure. For the studied range of conditions, we found that particle densities increase with particle diameter and entrance pressure. We measured areal particle densities of up to 4 × 10^−4^ to 1.9 × 10^−2^ particles per µm^2^ depending on particle diameter. The effective area of the focus is the region of the focus that is intense enough to produce measurable diffraction from a single particle. If we assumed that the nominal focus area [*i.e.* π(FWHM/2)^2^] matched the area of the focus region that is intense enough for producing measurable diffraction from a single particle, we would predict lower hit ratios (0.0003–37% for FWHM of 0.1–5 µm) than those that were in fact reached (between 0.8–79%) during past XFEL single-particle imaging experiments (Hantke *et al.*, 2014 ▸; Schot *et al.*, 2015 ▸; Reddy *et al.*, 2017 ▸; Daurer *et al.*, 2017 ▸). In fact, the effective focus area depends on many, mostly poorly known, variables, such as the intensity distribution in the interaction region, the beamline background, the detector response and the particle’s structure and orientation. For two XFEL single-particle imaging data sets (Hantke *et al.*, 2014 ▸; Daurer *et al.*, 2017 ▸) distributions of X-ray beam intensities of hits were determined from the diffraction data. In both cases the data showed that most diffraction patterns originated from weak hits at X-ray intensities far below half-maximum beam intensity (Hantke *et al.*, 2014 ▸; Daurer *et al.*, 2017 ▸). This means that for those experiments the effective focus area was considerably larger than the nominal focus area. We would expect the opposite for data acquired on much smaller particles under the same conditions.

For very high hit ratios the superposition of strong and spurious hits in a single diffraction pattern may present a problem (Hantke *et al.*, 2014 ▸). Therefore, moderate hit ratios (10–20%), as have been reached with the Uppsala injector for relatively large particles (100–500 nm), seem presently acceptable. Yet, the drastically lower particle densities for smaller particles, such as proteins, call for dedicated injector development.

Our results suggest that one possible strategy for reaching higher particle densities would be to increase the aerodynamic lens entrance pressure. But high aerodynamic lens entrance pressures are typically associated with high gas load on the sample chamber and interfere with vacuum requirements for X-ray detectors and other beamline equipment. In addition, high gas pressures in the focus can also cause undesired X-ray background noise. These problems could be mitigated to some extent by incorporating a differentially pumped shroud around the injector.

## 4. Conclusion   

Our results demonstrate the power of Rayleigh-scattering microscopy for tracking and sizing of focused aerosol particles. We anticipate that our characterization of the Uppsala injector will be useful to optimize data rates and data quality in future XFEL single-particle imaging experiments and will guide the development of new injectors in particular for small particles. New injection strategies are needed for reaching high particle densities, also for small particles, and unlocking the potential of femtosecond imaging on single molecules and chemical complexes. Furthermore, we envision that our Rayleigh-scattering-microscopy method will find application in other fields that employ focused aerosol beams, such as mass spectrometry, particle deposition and three-dimensional printing techniques.

## 5. Methods   

### 5.1. Experimental setup   

The stream of injected particles exiting from the tip of the aerodynamic lens was intersected with the single/dual-pulsed frequency-doubled Nd:YAG laser (Quantel Evergreen 25100, 532 nm wavelength). The laser provided pulse energies of up to 117 mJ resulting in peak intensities of 149 mJ mm^2^ at 7 ns pulse duration. The laser beam was focused by a plano-convex lens with 20 cm focal length to 0.6 mm FWHM at the intersection point with the particle beam. The laser beam was coupled into the experimental chamber through a glass window and was redirected three times by three consecutive mirrors, two before and one after the interaction point. Stray light was reduced by coupling the laser after the final mirror into a conical beam dump. For every measurement, the selection of pulse energy and neutral density absorption filter were optimized in order to obtain maximal signal while avoiding overexposure of the camera.

Particles were imaged with a microscope that is comprised of a stationary 2× objective lens (NA = 0.055, 91 µm depth of focus), placed inside the experimental chamber, and a motorized zoom lens (Navitar 12× UltraZoom) and CMOS camera (Hamamatsu Orca-Flash4.0 V2) on the outside. The optical axis of the microscope was aligned vertically with respect to the particle beam such that the angle between its optical axis and the laser-beam axis measured 25°.

The camera had 2048 × 2048 pixels, each with a sensitive area of 6.5 × 6.5 µm. The quantum efficiency was 0.8 and pixels were saturated at a signal of about 30 700 photons. The camera was operated at room temperature. Frames were acquired at a rate of 15 Hz in synchronization with the laser pulses. From 500 exposures we measured a mean photon background of 0.12 photons per pixel and per mJ of pulse energy at dark-noise fluctuations of, on average, 1.3 photons per pixel. The positional resolution in the image plane of 1.14 µm was calculated on the basis of the camera’s pixel spacing and the microscope lens’ configuration.

### 5.2. Image processing   

From the camera frames, peaks were detected and analyzed in a data analysis pipeline (https://github.com/mhantke/spts). For peak picking, two differently blurred versions of the image were generated using Gaussian kernels. The blur parameter σ was 0.03 and 0.06 pixels for the two versions. The difference image of the two blurred images was thresholded and a peak was assigned to each isolated cluster of pixels with values above a manually set threshold. For every measurement the threshold was adjusted manually to a value well above background fluctuations to minimize the selection of spurious peaks. From each peak, the particle position was determined by calculating the center of mass of the selected pixels. Back reflection resulted in the appearance of an additional faint peak at a constant displacement with respect to the main peak. We identified these spurious peaks and excluded them from further analysis. The brightness of each peak was determined by integrating the measured pixel values up to a radial distance of 10 pixels from the peak position. Peaks that were closer than 21 pixels apart were excluded from any further analysis.

### 5.3. Intensity calibration   

We injected suspensions of monodisperse polystyrene sphere size standards (Fischer Scientific, NIST-traceable size standard) to establish the relationship of peak brightness to particle diameter. The diameters of the size standards were 41 ± 4, 60 ± 4, 70 ± 3, 81 ± 3, 100 ± 3 and 120 ± 3 nm, and their variation coefficients were n.a., 17, 10.4, 11.7, 7.8 and 3.6%, respectively. The brightness of each particle was rescaled to its illumination using the beam profile, which we measured with a screen in a separate measurement. Calibration results are shown in Fig. 2 ▸(*c*) and are in agreement with the power law for Rayleigh scattering.

### 5.4. Model for particle acceleration   

Particle acceleration was modeled by calculating the drag of spherical particles in a freely expanding jet. The flow through the exit orifice (1.5 mm in diameter) forms a laminar (Re ≃ 1–100) continuous (Kn ≃ 10^−2^–10^−1^) flow field. Aerosol particles (40–500 nm in diameter) are about four orders of magnitude smaller than the orifice. Therefore, the Knudsen number for particle drag is about four orders of magnitude larger (Kn ≃ 10^2^–10^3^) and the flow field that is responsible for particle drag is governed by the laws of free molecular flow. We used the formula that estimates the spatial evolution of the Mach number at the centerline of a free jet (Ashkenas & Sherman, 1966 ▸; Dahneke & Cheng, 1979 ▸) to estimate the gas-flow field. The drag force was computed using a semi-empirical formula (Henderson, 1976 ▸), which is accurate for spherical particles at free-molecular-flow conditions. Gas velocity and the local state parameters of gas pressure, density and temperature were derived from the Mach number. Particles were inserted in the model system at a given initial velocity and propagated in the one-dimensional gas flow in an iterative scheme with dynamic adjustment of the step size to ensure accurate results while keeping computation time low.

For the ratio of effective and physical orifice diameter the best fit suggested a value of 0.880. Ashkenas & Sherman (1966 ▸) measured a value of 0.943 for a thin-plate orifice of similar dimensions to ours at nozzle Reynolds numbers above 500. The Reynolds numbers relevant for the study here are lower (Re ≃ 1–100). As the boundary layer at the orifice increases with decreasing Reynolds number the relatively smaller effective orifice diameter that we obtain is expected.

### 5.5. Sample preparation and aerosolization   

NIST-traceable polystyrene calibration spheres with diameters between 40 and 500 nm in an aqueous solution of 10^11^ particles ml^−1^ were used for the measurements. The nanospheres were aerosolized from the jet breakup of a 1.5 µm liquid jet from a GDVN running with 1–2 µl min^−1^ flow rate. With these conditions, between two to five particles were observed per frame on the camera, depending on particle diameter and injector pressure.

## Supplementary Material

Figures S1 and S2. DOI: 10.1107/S2052252518010837/ec5009sup1.pdf
