# Supplementary material for: Rayleigh-scattering microscopy for tracking and sizing nanoparticles in focused aerosol beams
Source: IUCrJ. 2018 Sep 11;5(Pt 6):673–80. doi: 10.1107/S2052252518010837 (PMC6211534; doi:10.1107/S2052252518010837)
Supplement: Supplementary file 1 [file m-05-00673-sup1.pdf]

IUCrJ

Volume 5 (2018)

Supporting information for article:

Rayleigh-scattering microscopy for tracking and sizing nanoparticles in focused aerosol beams

Max F. Hantke, Johan Bielecki, Olena Kulyk, Daniel Westphal, Daniel S. D. Larsson, Martin Svenda, Hemanth K. N. Reddy, Richard A. Kirian, Jakob Andreasson, Janos Hajdu and Filipe R. N. C. Maia

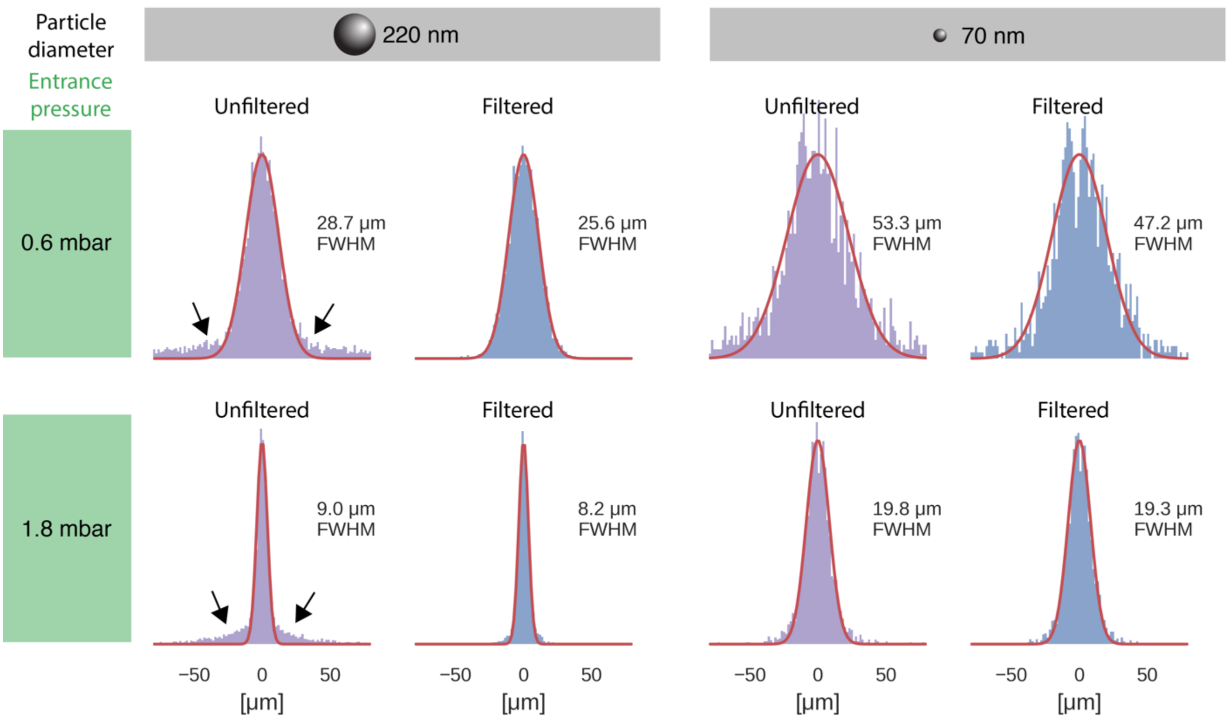

Figure S1 **Data filter for excluding particle clusters.** The histograms show the distributions of aerosol-particle positions in the particle beam focus measured in transverse direction with respect to the particle beam axis. The solid red lines represent Gaussian fits to the histograms. Particles were aerosolised from a suspension of polystyrene sphere size standards with nominal diameters of 200 nm (left panel) and 70 nm (right panel). The particles were injected at entrance pressures of 0.6 mbar (top row) and 1.8 mbar (bottom row). For comparison histograms were generated before (purple histograms) and after (blue histograms) excluding particles that exceed the respective nominal diameter by more than 10 nm. We believe that large particles that we excluded are a result of particle-aggregation. We observe that for all histograms the distributions of the size-filtered data are more narrow (i.e. smaller FWHM) than the unfiltered distributions. For the data set obtained from the 220 nm polystyrene spheres we find that the unfiltered distributions do not match the Gaussian model (see arrows that point to the mismatch at the wings). The distributions of monodisperse subpopulations are well approximated by the Gaussian model.

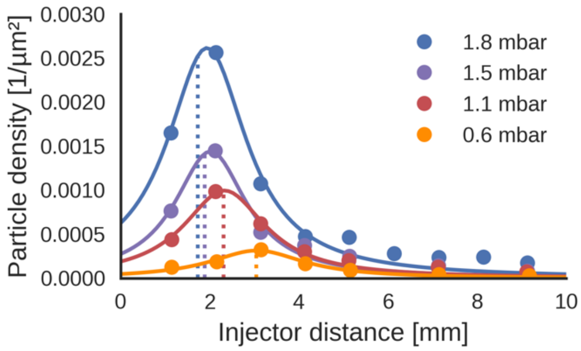

Figure S2 **Particle beam density.** Particle number density as a function of the distance from the injector exit nozzle at four different aerodynamic lens entrance pressures 0.6, 1.1, 1.5, and 1.8 mbar (see legend). The data were measured for injected polystyrene spheres of 70 nm and particle densities were normalized to a particle solution with a concentration of 10^{12} particles per mL and a flow rate of 1 μL/min. The values were approximated by intensities of a Gaussian beam profile (solid lines). The focus positions (Fig. 3a) are indicated by vertical dotted lines and these positions approximately match the positions from the Gaussian beam density fit.
